# Plasticity of left perisylvian white-matter tracts is associated with individual differences in math learning

**DOI:** 10.1007/s00429-014-0975-6

**Published:** 2015-01-21

**Authors:** Dietsje Jolles, Demian Wassermann, Ritika Chokhani, Jennifer Richardson, Caitlin Tenison, Roland Bammer, Lynn Fuchs, Kaustubh Supekar, Vinod Menon

**Affiliations:** 1 Department of Psychiatry and Behavioral Sciences, Stanford University School of Medicine, 1070 Arastradero Road, Suite 220, Palo Alto, CA 94304 USA; 2 Department of Neurology and Neurological Sciences, Stanford University School of Medicine, Stanford, CA USA; 3 Program in Neuroscience, Stanford University School of Medicine, Stanford, CA USA; 4 Symbolic Systems Program, Stanford University School of Medicine, Stanford, CA USA; 5 Department of Radiology, Center for Quantitative Neuroimaging, Stanford University School of Medicine, Stanford, CA USA; 6 Department of Education and Child Studies, Leiden University, Wassenaarseweg 52, 2333 AK Leiden, The Netherlands; 7 Department of Radiology, Brigham and Women’s Hospital and Harvard Medical School, Cambridge, MA USA; 8 Department of Special Education, Vanderbilt University, Nashville, TN USA; 9 Athena EPI, INRIA Sophia Antipolis - Méditerranée, 2004 route des Lucioles, 06902 Sophia Antipolis, France

**Keywords:** Academic, Arithmetic, Diffusion tensor imaging (DTI), Learning, Plasticity, Superior longitudinal fasciculus

## Abstract

Plasticity of white matter tracts is thought to be essential for cognitive development and academic skill acquisition in children. However, a dearth of high-quality diffusion tensor imaging (DTI) data measuring longitudinal changes with learning, as well as methodological difficulties in multi-time point tract identification have limited our ability to investigate plasticity of specific white matter tracts. Here, we examine learning-related changes of white matter tracts innervating inferior parietal, prefrontal and temporal regions following an intense 2-month math tutoring program. DTI data were acquired from 18 third grade children, both before and after tutoring. A novel fiber tracking algorithm based on a White Matter Query Language (WMQL) was used to identify three sections of the superior longitudinal fasciculus (SLF) linking frontal and parietal (SLF-FP), parietal and temporal (SLF-PT) and frontal and temporal (SLF-FT) cortices, from which we created child-specific probabilistic maps. The SLF-FP, SLF-FT, and SLF-PT tracts identified with the WMQL method were highly reliable across the two time points and showed close correspondence to tracts previously described in adults. Notably, individual differences in behavioral gains after 2 months of tutoring were specifically correlated with plasticity in the left SLF-FT tract. Our results extend previous findings of individual differences in white matter integrity, and provide important new insights into white matter plasticity related to math learning in childhood. More generally, our quantitative approach will be useful for future studies examining longitudinal changes in white matter integrity associated with cognitive skill development.

**Electronic supplementary material:**

The online version of this article (doi:10.1007/s00429-014-0975-6) contains supplementary material, which is available to authorized users.

## Introduction

Understanding how plasticity of white matter tracts contributes to learning is a fundamental question in developmental cognitive neuroscience, and lies at the foundation of understanding complex interactions between brain structure and function. The long-range white matter pathways linking parietal, temporal and prefrontal cortex undergo protracted developmental changes, which may interact with cognitive and academic skill development by regulating the speed and synchrony of signal transmission between brain regions. Almost all prior studies in children have examined white matter organization using a cross-sectional approach (e.g., Tamnes et al. 2010; Barnea-Goraly et al. 2005b; Lebel et al. 2008; Asato et al. 2010; Snook et al. 2005). While a few recent studies examined longitudinal changes in white matter with age (e.g., Yeatman et al. 2012; Lebel and Beaulieu 2011; Giorgio et al. 2010), little is known about how learning and experience influence white matter organization in children. One important challenge for studies measuring longitudinal changes associated with learning is the identification of tracts across multiple time points, a process that is often subjective and poorly reproducible. Better quantitative characterization of learning-related changes in key white matter tracts, and their relation with individual differences in performance improvement, is important as it may improve our understanding of the mechanisms by which brain plasticity supports academic skill development. Here, we use a 2-month arithmetic training paradigm in combination with novel quantitative diffusion tensor imaging (DTI) analysis methods to examine white matter plasticity associated with mathematics learning in a tight age range of 7- to 9-year-old children.

Mathematics is an important domain of academic skill development. However, compared to reading, interventions in mathematics have received little attention. A better understanding of mathematical skill acquisition is warranted since early math proficiency is hypothesized to be a critical factor in driving future life success (Parsons and Bynner 2005; Gross et al. 2009; Ritchie and Bates 2013). Children show significant individual differences in their ability to perform simple arithmetic operations. Importantly, differences in conceptual understanding and procedural knowledge during the early stages of learning can lead to subsequent difficulties in academic skill acquisition during later grades (Siegler 2003; Fuchs et al. 2010). Intervention and tutoring programs have been proposed to ameliorate deficits in basic arithmetic problem solving, including instruction on counting strategies, building understanding of part–whole relationships, as well as drill and practice to promote retrieval of math facts from long-term memory (Fuchs et al. 2008, 2010). Despite the effectiveness of such tutoring programs, no studies have examined the underlying white matter pathways that support math learning.

Neurocognitive theories suggest that arithmetic processing involves distinct verbal, visual and quantity representations, which engage distributed interacting brain areas (Menon 2013; Arsalidou and Taylor 2011; Kaufmann et al. 2011). A central role has been assigned to the posterior parietal cortex, specifically the intraparietal sulcus (IPS), which is thought to be involved in numerical quantity processing (Dehaene et al. 2003; Ansari 2008; Bugden et al. 2012), the supramarginal gyrus, which contributes to arithmetical computation and working memory (Rivera et al. 2005; Menon et al. 2000; Zago and Tzourio-Mazoyer 2002), and the angular gyrus, which has been implicated in verbally mediated retrieval (Dehaene et al. 1999, 2003). Furthermore, ventral temporal–occipital regions are often engaged, related to the visual representation of number symbols (Dehaene and Cohen 1995; Lyons and Ansari 2009; Holloway et al. 2013). Moreover, depending on problem complexity, prefrontal cortex regions important for working memory and executive control processes are recruited (Arsalidou and Taylor 2011), especially in children, who require additional resources to compensate for inefficient and less-automated problem-solving strategies (Rivera et al. 2005; Ansari 2008; Houde et al. 2010). The integration of cognitive processes across multiple brain systems is thought to be of critical importance for math problem solving in children (Supekar and Menon 2012; Rosenberg-Lee et al. 2011), and requires information transfer across white matter tracts that link parietal, temporal and prefrontal cortices.

A number of cross-sectional studies, some in children and others in adolescents, have suggested a relation between mathematical performance and the integrity of association pathways connecting inferior parietal, frontal and temporal lobes (van Eimeren et al. 2008; Tsang et al. 2009; Van Beek et al. 2013; Matejko et al. 2012; Navas-Sanchez et al. 2013). For example, in children, a positive correlation has been reported between white matter integrity and approximate arithmetic ability (Tsang et al. 2009), as well as exact addition and multiplication performance (Van Beek et al. 2013), in a subsection of the left superior longitudinal fasciculus (SLF) linking frontal with posterior parietal cortex. A similar brain–behavior correlation has been found for college students in left parietal white matter regions including the SLF (Matejko et al. 2012). Moreover, a recent study in math-gifted children revealed increased white matter integrity in bilateral fronto-striatal and fronto-parietal tracts compared to control children (Navas-Sanchez et al. 2013). Importantly, this effect remained after IQ differences were controlled. At the other end of the spectrum, children who demonstrate difficulties in mathematics, including children with developmental dyscalculia (Rykhlevskaia et al. 2009; Kucian et al. 2013), fetal alcohol spectrum disorder (Lebel et al. 2010), and velocardiovascular syndrome (Barnea-Goraly et al. 2005a) tend to show reduced white matter integrity in tracts connecting frontal, parietal and temporal cortices, including the SLF and the inferior longitudinal fasciculus.

While these studies suggest a positive relation between white matter integrity and math ability, there are inconsistencies in the regional specificity of the findings, which is likely due to differences in methodology, cognitive measures and participant age across studies. Nevertheless, most studies point to white matter tracts connecting or traversing through the inferior parietal lobule and IPS. Although the taxonomy of inferior parietal connections is still under discussion (Axer et al. 2013; Schmahmann and Pandya 2006; Dick and Tremblay 2012), the evidence points to three major tract bundles connecting inferior parietal regions with the temporal and frontal lobes (Zhang et al. 2010): (1) a fronto-parietal tract, composed of the anterior segment of the SLF which connects the parietal and frontal lobes coursing superiorly to the Sylvian fissure (Makris et al. 2005; Catani et al. 2005); (2) a parieto-temporal tract, composed of the posterior segment of the SLF (Catani et al. 2005) and the dorsal segment of the inferior longitudinal fasciculus (Makris et al. 1999), connecting the parietal and temporal lobes and coursing inferior to the Sylvian fissure; and (3) a fronto-temporal section of the SLF, or arcuate fasciculus, which connects the frontal and temporal lobes traversing the parietal lobe posterior to the Sylvian fissure (Makris et al. 2005; Catani et al. 2005). It remains to be investigated to what extent math tutoring can produce tissue changes in these white matter tracts and, more importantly, whether these changes are associated with behavioral improvement.

Here, we examine white matter changes associated with math learning using a well-validated, one-on-one arithmetic tutoring program (Fuchs et al. 2009). Our study focused on a narrow age range of children all in grade 3 (ages 7–9), a developmental stage important for learning and mastering basic arithmetic facts (Fuchs et al. 2009). In a previous study, we showed that tutoring-related performance gains could be predicted based on brain structure and function before the start of the tutoring (Supekar et al. 2013). In the present study, we focus on plasticity of white matter tracts with tutoring. Based on findings of positive correlations between math ability and white matter integrity in previous cross-sectional DTI studies, we predicted that white matter integrity would increase with tutoring and, furthermore, that children showing greater white matter integrity changes with tutoring would show greater performance gains.

We focused specifically on the three sections of the SLF, innervating the inferior parietal, frontal and temporal regions important for math, i.e., the fronto-parietal section of the SLF (SLF-FP), the fronto-temporal section of the SLF (SLF-FT), and the parieto-temporal section of the SLF (SLF-PT). We used a novel automated atlas-based tracking algorithm based on a White Matter Query Language (WMQL) that allowed precise and consistent delineation of fiber tracts in each individual (Wassermann et al. 2013). Individual fiber tracts were then used to construct population-specific probabilistic maps for each tract, from which we extracted mean fractional anisotropy (FA), a quantitative measure of white matter organization which is influenced by changes in integrity (Beaulieu 2002; Basser 1995). This approach allows comparisons of the exact same tract within and between individuals in a manner robust to errors in the fiber tracking of each individual (Hua et al. 2008; Zhang et al. 2010). Notably, the accuracy of this automated technique has shown to be comparable to manual labeling (Wassermann et al. 2013), but is less time-consuming and allows easier replication. The main advantage of the WMQL framework is the elimination of the operator-specific intra- and inter-subject inconsistencies in tract delineation. These inconsistencies have been argued to be a major source of variability in tractography-based studies (Zhang et al. 2010). Previous efforts to perform tractography-based longitudinal studies tackle this problem with semi-automated methods (Lebel and Beaulieu 2011), which are user intensive and difficult to reproduce. WMQL, a freely available tool,^1^ provides an innovative solution to this problem combining a publicly available parcellation of gray and white matter regions (Desikan et al. 2006) with tract descriptions written in a well-defined query language, thus tackling the operator-based variability in tractography delineation and providing clear means to reproduce the tract-extraction process and the statistical analyses.

## Material and methods

### Overall study design

The study was conducted in four phases: (1) initial neuropsychological assessments, (2) Time 1 brain imaging session (3) math tutoring, and (4) Time 2 brain imaging session (cf. Supekar et al. 2013). Each brain imaging session included DTI as well as task-related functional magnetic resonance imaging (fMRI) scans that were used to measure arithmetic problem solving. After successful completion of the first brain imaging session (Time 1), children started a 2-month (8 weeks) math tutoring program that had been shown to be effective in school-based studies of children with math difficulties (Fuchs et al. 2009) and students with otherwise low math skill (Fuchs et al. 2013). Children received tutoring three times per week for approximately 40–50 min per session. Tutoring was carried out one-on-one and involved both conceptual instruction and speeded procedural practice on simple addition and subtraction problems, which gradually increased in difficulty. By the end of tutoring, students learned addition problems that summed up to 18 and the complementary subtraction problems. For an overview of the tutoring procedures, see Online Resource 1. At the end of the 8 weeks, the children returned for a second brain imaging session (Time 2). The overall design is the same as in our previous study in which we focused on Time 1 predictors of learning using gray matter and intrinsic functional connectivity (Supekar et al. 2013). The present study builds on our previous study and focuses on changes in white matter pathways between Time 1 and Time 2, but uses a slightly different sample based on the availability and quality of DTI data from each child.

### Participants

To minimize the effects of age-related changes and to focus on an important developmental phase for math skill acquisition, we recruited children in grade 3 only. From an initial group of 22 children, 2 children were excluded because of poor DTI quality and 2 children were excluded because their performance gains were greater than 3 SDs from the group mean. The final group included 18 children (ages 7.7–9.1; 11F/7M). None of the included children were left handed (as assessed using the Edinburgh handedness test), or had a history of neurological or psychiatric disorders. Informed consent was obtained from the legal guardian of the child, and study protocols were approved by the Stanford University Institutional Review Board. All participants underwent a comprehensive battery of standardized neuropsychological assessments. IQ was determined using the Wechsler Abbreviated Scale of Intelligence (WASI) (Wechsler 1999). Academic achievement in reading and mathematics was assessed using the Wechsler Individual Achievement Test-Second Edition (WIAT-II) (Wechsler 2001) and KeyMath3 (Connolly 2007). Working memory was assessed using the Working Memory Test Battery for Children (WMTB-C) (Pickering and Gathercole 2001). Mean scores and standard deviations are presented in Supplementary Table 1 (Online Resource 2).

### Tutoring outcome measures: addition and subtraction verification tasks

Math problem solving was assessed before and after tutoring using addition and subtraction verification tasks obtained during a separate fMRI scanning session. There were at least two addition and two subtraction runs per session, which included 12 arithmetic problems each. For the analyses presented here, we focused on behavioral data from the first two addition and two subtraction runs that were available for analyses. The arithmetic problems were presented horizontally in green lettering on a black background. Half the math problems were correct (e.g., 3 + 4 = 7), and the other half deviated from the correct answer by ±1 or ±2 (e.g., 3 + 5 = 7). Only single-digit problems were included, and problems with 1 or 0 were excluded. In the addition task, the larger operand was equally likely to appear in the first or second position. Each trial started with a fixation asterisk for 0.5 s. The arithmetic problem was presented for up to 9.5 s, during which the child had to make a response. Participants used a response box to indicate “1” if the problem was correct and “2” if the problem was incorrect. After the response, the problem disappeared from the screen, and a black screen appeared for the remainder of the 9.5 s. Non-arithmetic control problems were randomly interspersed with arithmetic trials but are not analyzed here. In this task two numbers were presented in the format of “n = m” where n and m were numbers between 0 and 25. Half the control problems were presented as correctly matched pairs (i.e., 3 = 3) and the other half deviated from the true answer by ±1 or ±2 (i.e., 3 = 5). Children were told to indicate “1” if the two numbers were the same or “2” if the two numbers were different.

Analyses of performance improvement were conducted using repeated measures ANOVAs. To derive an aggregate measure that captures all performance changes across the addition and subtraction verification tasks, we computed an “efficiency score”, based on Salthouse and Hedden’s (2002) definition of efficiency. Efficiency change was calculated as follows: (1) for each of the four behavioral measures separately (i.e., accuracy and reaction time (RT) for addition and subtraction problems), we calculated z-scores based on the group mean and standard deviation across Time 1 and Time 2; (2) we averaged the z-scores for accuracy and the inverted z-scores for RT for each participant within each time-point; (3) efficiency change was calculated by subtracting Time 1 efficiency from Time 2 efficiency (Supekar et al. 2013). For these analyses, median RT was calculated across correct trials only. Invalid trials (during which button presses other than 1 or 2 were recorded) were treated as missing values in the analysis. If there were more than 2 invalid trials in a run, we used the subsequent run for that participant (which was the case in 4 subjects).

### DTI methods

#### DTI acquisition

The DTI pulse sequence was a diffusion-weighted single-shot spin-echo, echo planar imaging sequence with ramp sampling (TE = 81.5 ms; TR = 9.05 s; field of view = 220 mm; matrix size = 128 × 128; partial k-space acquisition, chemsat-based fat suppression; maximum gradient strength used for diffusion-weighting was 50 mT/m). We acquired 63 axial, 2-mm-thick slices (no skip) for two *B* values, *B* = 0 and *B* = 900 s/mm^2^; 3 repeats. The high *B* value was obtained by applying gradients along 23 different diffusion directions that were evenly spread over one hemisphere.

#### DTI preprocessing

Diffusion tensor imaging data were preprocessed using a custom program based on normalized mutual information that removed eddy current distortion effects and determined a constrained nonrigid image registration of all the diffusion images (Bammer et al. 2002). The six elements of the diffusion tensor were determined by multivariate regression (Basser 1995; Basser and Pierpaoli 1996). For each subject, the baseline non-diffusion-weighted (*B* = 0) images were coregistered to the T1-weighted 3-D SPGR anatomical images using a mutual information 3-D rigid-body coregistration algorithm from SPM5. Several anatomical landmarks, including the anterior commissure (AC), the posterior commissure (PC), and the midsagittal plane, were identified by hand in the T1 images. With these landmarks, we computed a rigid-body transform from the native image space to the conventional AC–PC-aligned space. The DTI data were then resampled to this AC–PC-aligned space with 2 mm isotropic voxels using a spline-based tensor interpolation algorithm (Pajevic et al. 2002), taking care to rotate the tensors to preserve their orientation with respect to the anatomy (Alexander et al. 2001).

DT images were estimated from their corresponding diffusion-weighted acquisitions using the mrDiffusion tool (http://white.stanford.edu/newlm/index.php/MrDiffusion). The same tool was used to compute an FA image for each scan as well as a whole-brain tractography (Basser et al. 2000).

#### DTI tract analysis

To reliably identify the SLF components across children at multiple time points, we used an atlas-based procedure that segmented fiber tracts in each subject’s native space based on the WMQL (Wassermann et al. 2013). This technique allows the automated extraction of tract-specific measures from the whole-brain tractography of each scan in each person without the need to manually delineate the tracts (Hua et al. 2008). Fiber tracking was performed in individual space, similar to manual tractography, yet the approach had the advantage and precision of using a query language that involves precise specification of (1) the tract endpoints, (2) the anatomical structures that the tract traverses through, and (3) the position relative to other anatomical structures (Wassermann et al. 2013). Individual fiber tracts were combined to create population-based probabilistic tract maps, from which we extracted mean FA values in each participant, at each time-point. The reason for creating probabilistic maps rather than using individually traced tracts was twofold. First, we wanted to avoid spurious FA changes due to fact that slightly different fibers were traced at each time-point. Second, tract definition in some children was more successful than in others due to noise in the data, a common issue in pediatric DTI studies. Next, we averaged FA across the tract in order to compute overall tissue differences on the tracts without making the assumption (as in voxel-wise or along-the-tract analyses) that the change in white matter tissue occurred in a specific tract section. By weighting the FA of each voxel by its probability, greatest emphasis was placed on the core of each tract (i.e., the regions that are most consistent across participants), which is least likely influenced by partial voluming effects and crossing fibers (Hua et al. 2008).

**Fig. 1:**
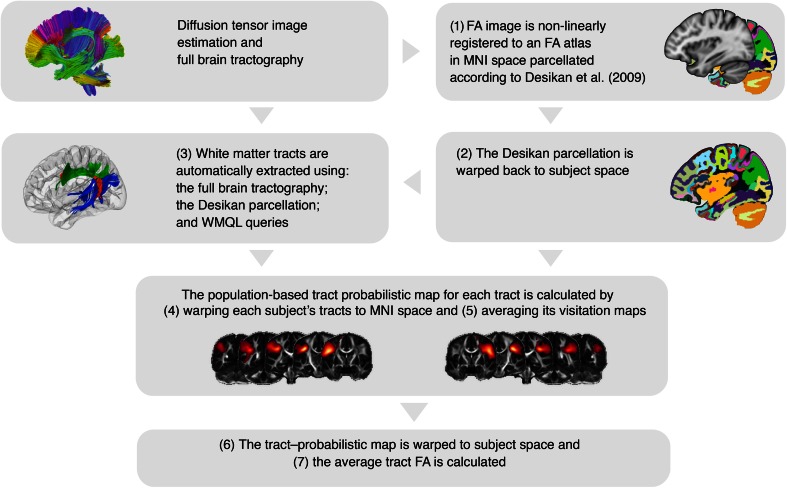
DTI processing steps. The input to the pipeline include diffusion tensor imaging (DTI) data, a fractional anisotropy (FA) template with a Desikan et al. (2006) parcellation superimposed, and a set of queries in White Matter Query Language (WMQL) describing the sections of the superior longitudinal fasciculus (SLF) to be obtained. The pipeline involves an initial step for each subject estimating the DTI image and calculating from this image the FA map and a full-brain tractography. Next, child-specific probabilistic template maps are computed using the following procedures: (1) the FA image is registered to the Montreal Neurological Institute (MNI) template and (2) the Desikan parcellation is warped back to the subject using the inverse transformation map. Then, (3) the WMQL and the Desikan parcellation are used to obtain the sections of the SLF for each subject from the full-brain tractography, and (4) these tracts are warped to the template space to (5) generate the population-based tract probabilistic maps by averaging the visitation maps of all subjects. Finally, (6) these probabilistic maps are warped back to subject space and (7) used to compute the average FA of each tract for each subject and perform statistical analysis

An overview of DTI analysis steps is displayed in Fig. 1. For each of the tracts of interest, a measure of white matter integrity was extracted by (1) non-linearly registering the FA images to MNI space using the template by Zhang et al. (2011) and the Advanced Normalization Tools (ANTS), which implement the algorithms by Avants et al. (2008); (2) transforming Desikan atlas labels (Desikan et al. 2006) to individual subject space using the inverse transformation map; (3) extracting tracts in individual subject space using the Desikan parcellation and the WMQL; (4) warping the scan-specific tracts to MNI space using the obtained deformation map; (5) computing binary visitation maps for each tract and averaging the visitation maps for each tract across participants; (6) transforming the probabilistic maps to each individual’s space using the inverse transformation map; (7) extracting a single FA value for each tract, participant, and scanning session using these tract probabilistic maps, which were thresholded at 0.01 to omit regions scarcely visited by the tract on most subjects. For this, we computed the weighted average FA value for each tract and each scan as (Hua et al. 2008):

$${\text{FA}}_{\text{tract}} = \frac{{\varSigma_{i} {\text{FA}}_{i} \times \Pr_{i} }}{{\varSigma_{i} \Pr_{i} }},$$

where *i* is the index of each voxel in the image, FA_*i*_ is the FA value at voxel *i* and Pr_*i*_ is the value of the tract probabilistic map at voxel *i*. In this manner, voxels that belong to core regions of tracts, which are reproducible in the sample, have larger weighting for tract-specific quantification.

The reliability of tract identification and the FA_tract_ measure were assessed separately. To assess the reliability of tract identification across time-points, we measured the within-subject overlap of each of the three SLF components. To avoid an arbitrary discretization of the tracts bundles obtained through WMQL, we interpreted each bundle as point densities in 3D and quantified the overlap between these densities using the Bhattacharyya coefficient (Rao 1952). This coefficient takes a value of 1 for perfect overlap between tracts and 0 for no overlap. As with Cohen’s Kappa, we consider a value of 0.8 an almost perfect overlap. We assessed the reliability of the FA_tract_ measure independently of the tutoring, using a tract that is not expected to change with tutoring: the cortico-spinal tract (CST). We analyzed the reliability of the FA_tract_ measure on the CST by extracting the CST in all subjects, computing the measure, and then using the intra-class correlation of type ICC (3,1) (Landis and Koch 1977) with the two time points as different raters.

Repeated measures ANOVAs were used to examine FA changes in the SLF sections after tutoring. In addition, we performed correlation analyses to examine the relation between FA changes and changes of performance. To reduce the number of comparisons for the brain–behavior correlations, we used efficiency change scores that captured changes of accuracy and RT for both of the tasks (Salthouse and Hedden 2002; Supekar et al. 2013). Participants with FA changes larger than 3 SDs were considered outliers and removed from the specific analyses.

## Results

### SLF tract identification

**Fig. 2:**
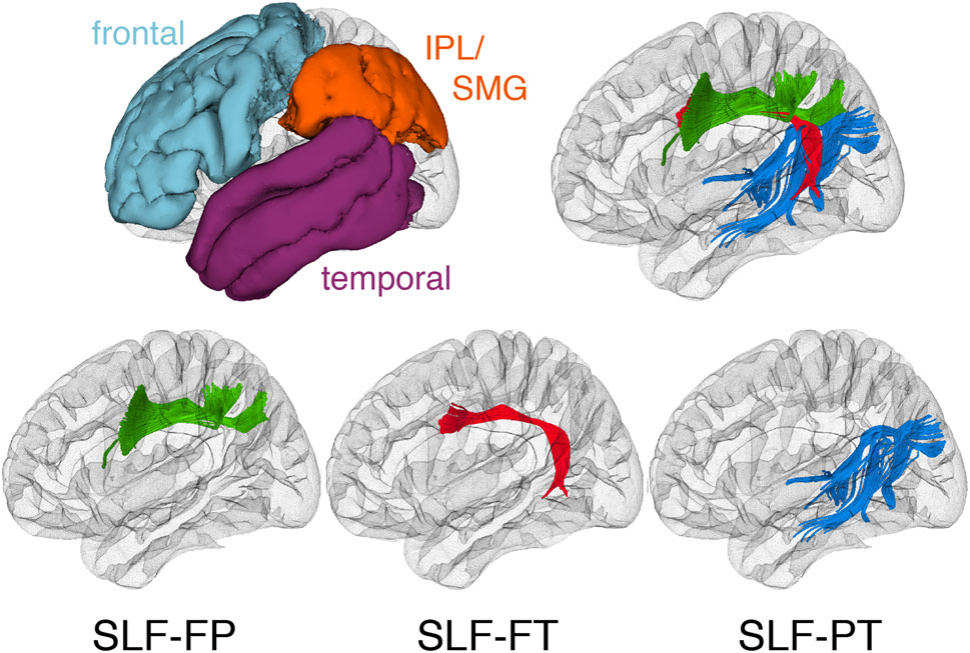
Tract definitions. On the *upper left*, the three regions used to generate queries for the white matter tracts: the frontal lobe in *cyan*, the temporal lobe in *purple*, and the supramarginal gyrus (SMG) and inferior parietal lobule (IPL) in *orange*. For illustration purposes, the extracted tracts of a single subject are shown on the *upper right* and separate in the *second row*. Using a nomenclature based on Zhang et al. (2010), these tracts are defined as the fronto-parietal section of the superior longitudinal fasciculus (SLF-FP) in *green*, the fronto-temporal section of the superior longitudinal fasciculus (SLF-FT) in *red*, and the parieto-temporal section of the superior longitudinal fasciculus (SLF-PT) in *blue*

**Fig. 3:**
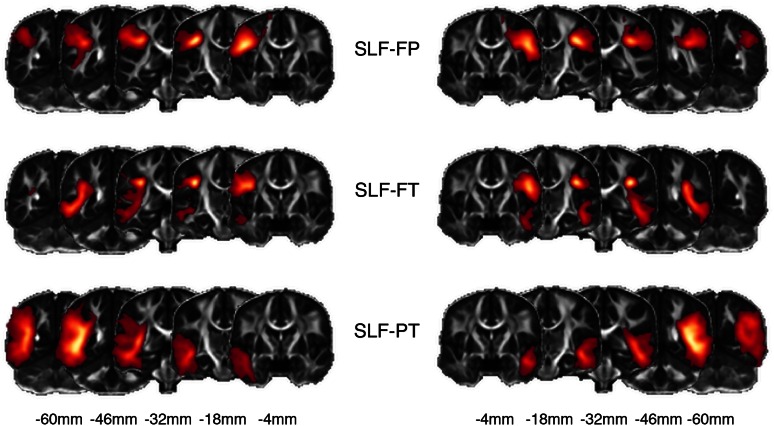
Probabilistic maps. For each one of the superior longitudinal fasciculus (SLF) sections obtained with the White Matter Query Language (WMQL), we created population-based probabilistic maps by averaging the visitation maps of each subject. From *top* to *bottom*, the fronto-parietal section of the SLF (SLF-FP), the fronto-temporal section of the SLF (SLF-FT), and the parieto-temporal section of the SLF (SLF-PT), superimposed on the fractional anisotropy (FA) template image. The *color* map indicates the highest probability of the fascicle traversing that area in space in *yellow* and the lowest in *red*

We used a novel approach based on the White Matter Query Language (Wassermann et al. 2013) to extract SLF tracts in each child. We reliably identified three major association tract bundles per hemisphere connecting fronto-parietal, fronto-temporal and parieto-temporal cortices (Catani et al. 2005, 2007; Makris and Pandya 2009; Makris et al. 2005). The tracts were defined as follows (see Online Resource 3 for the white matter query): (1) the SLF-FP (Fig. 2, in green) involved the fiber bundles contained in the frontal and parietal lobes connecting the frontal lobe with the supramarginal and inferior parietal convolutions. (2) The SLF-FT (Fig. 2, in red) involved the fiber bundles connecting the inferior frontal, middle frontal and precentral gyrus with the superior and middle temporal gyrus coursing through the parietal lobe, but not medial of the supramarginal gyrus (Makris and Pandya 2009; Catani et al. 2005, 2007). (3) The SLF-PT (Fig. 2, in blue) involved the fiber bundles contained in the temporal and parietal lobes connecting the temporal lobe with the inferior parietal convolutions. The obtained population-based tract probabilistic maps resulting from this process are shown in Fig. 3.

To assess the reliability of tract identification across subjects, we measured the within-subject overlap of the three SLF components across the two time points. The reliability of the tract-identification process was high. The maximum average overlap was found for the right SLF-PT (0.96 ± 0.06) and the minimum overlap was found for the left SLF-FP (0.90 ± 0.05). The detailed results are presented in Supplementary Fig. 1 (Online Resource 4). Moreover, the reliability of the FA_tract_ measure was also high. The ICC coefficient for the left CST was 0.92 (95 % confidence interval 0.74–0.98) and for the right CST 0.95 (95 % confidence interval 0.84–0.98).

### Performance gains with 8 weeks of math tutoring

**Fig. 4:**
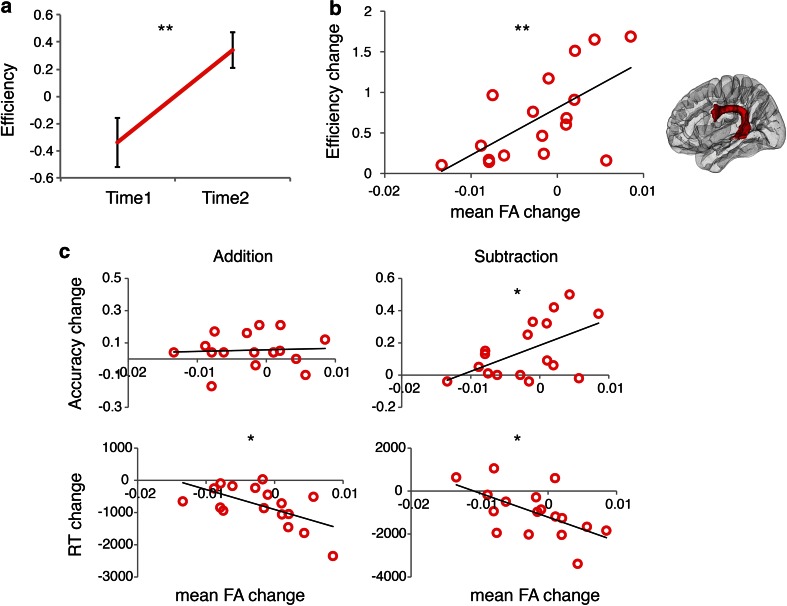
Performance gains are associated with FA changes in the left SLF-FT. **a** Changes of performance efficiency after math tutoring. Performance efficiency was assessed using a composite of standardized accuracy and median reaction time (RT) scores on addition and subtraction tasks (*n* = 18). *Error bars* represent standard error of mean. **b** Changes of performance efficiency were correlated with fractional anisotropy (FA) changes in the fronto-temporal part of the left superior longitudinal fasciculus (SLF-FT) (Time 2 − Time 1; *n* = 17). **c** FA changes were correlated with changes in three of the four sub-measures of performance change (i.e., accuracy on subtraction and RT on addition and subtraction tasks) that made up the efficiency score (Time 2 − Time 1; *n* = 17). The performance sub-measures were not significantly correlated amongst themselves. **p < 0.01, *p < 0.05

Performance efficiency significantly increased with math tutoring (*F*(1, 17) = 29.29, *p* < 0.001, *η*_p_^2^ = 0.633; Fig. 4a). Follow-up analyses indicated that for both the addition and the subtraction task, there were accuracy gains (addition: *F*(1, 17) = 6.22, *p* = 0.023, *η*_p_^2^ = 0.268; subtraction: *F*(1, 17) = 14.43, *p* = 0.001, *η*_p_^2^ = 0.459), as well as RT decreases (addition: *F*(1, 17) = 31.60, *p* < 0.001, *η*_p_^2^ = 0.650; subtraction: *F*(1, 17) = 6.16, *p* = 0.024, *η*_p_^2^ = 0.266).

### White matter integrity changes in left SLF-FT correlated with individual performance gains after tutoring

We first examined overall white matter changes following 8 weeks of math tutoring. Averaged across all subjects, there were no significant FA changes with tutoring in any of the tracts (all *p*s ≥ 0.179; Supplementary Table 2, Online Resource 2). Next, we examined whether changes in FA were correlated with individual differences in performance gain. The left SLF-FT was the only tract that showed a significant relation after Bonferroni correction for multiple comparisons (*r* = 0.630, *p* = 0.007, *n* = 17, 95 % confidence interval, bootstrapped from 1,000 samples: 0.184–0.887; Fig. 4b).

Because of partial spatial overlap between the three different tracts in each hemisphere, we performed post hoc partial correlation analyses to control for FA changes in adjacent tracts. We found that the correlation between efficiency change and FA change in the left SLF-FT was still significant when controlling for FA change in the left SLF-PT and SLF-FP (*r* = 0.607, *p* = 0.016, *df* = 13). Additional post hoc analyses were performed to examine the correlation between FA change and improvement in the four performance sub-measures that made up the composite efficiency score (i.e., accuracy and RT for addition and subtraction tasks). We found correlations with improvement in three of the four sub-measures (subtraction accuracy: *r* = 0.535, *p* = 0.027, *n* = 17; addition RT: *r* = −0.592, *p* = 0.012, *n* = 17; subtraction RT: *r* = −0.579, *p* = 0.015, *n* = 17) (Fig. 4c). The performance sub-measures were not significantly correlated amongst themselves (*p*s ≥ 0.072).

The only other tract in which behavioral improvement was associated with FA changes was the right SLF-FP, but this effect was only marginally significant (*r* = 0.466, *p* = 0.051, *n* = 18, 95 % confidence interval, bootstrapped from 1,000 samples: 0.066–0.747 s; Supplementary Fig. 2, Online Resource 4). Although the relation between efficiency change and FA change became more significant when controlling for FA change in the right SLF-FT and SLF-PT, it still did not survive Bonferroni correction (*r* = 0.574, *p* = 0.020, *df* = 14). Correlations with the other tracts did not reach significance (all *p*s ≥ 0.223; Supplementary Table 3, Online Resource 2). We conducted additional analyses for the SLF-PT excluding three subjects for whom the inferior-most sections of the temporal lobe were not scanned. Excluding these subjects did not change main effects (*p* = 0.875 and *p* = 0.804 for left and right SLF-PT, respectively) or brain–behavior correlations (*p* = 0.107 and *p* = 0.557 for left and right SLF-PT, respectively).

### White matter integrity of left SLF-FT was not associated with individual differences in math abilities before tutoring

Finally, we examined whether FA in the left SLF-FT, which showed a significant tutoring effect, also showed a relation with math abilities prior to tutoring. We found that FA in this tract was not significantly correlated with standardized measures of math abilities before tutoring, including Numerical Operations and Mathematical Reasoning subtests of the WIAT-II and the Numeration and Algebra subtests of KeyMath3, or with performance efficiency before tutoring (all *p*s ≥ 0.402; Supplementary Table 4, Online Resource 2).

## Discussion

The maturation of white matter pathways increases the speed and efficiency of information processing in the brain and is therefore an important factor in the development of cognitive and academic skills. Reciprocally, white matter maturation is also influenced by learning and skill acquisition. For example, it has been argued that myelination, which influences the speed and synchrony of neuronal firing, can be modified by impulse activity, thereby providing a mechanism to optimize information processing based on environmental demands (Fields 2005, 2008). Here, for the first time, we examined changes in white matter tracts associated with math skill acquisition in primary grade school children. We used a novel approach to create child-specific templates of the three major sections of the SLF: the fronto-parietal tract (SLF-FP), the fronto-temporal tract (SLF-FT) and the parieto-temporal tract (SLF-PT), hypothesized to be important for linking distributed brain areas involved in mathematical information processing. Importantly, our approach involved an automated fiber tracking algorithm based on the White Matter Query Language, thereby avoiding operator-specific intra- and inter-subject inconsistencies in tract delineation. For multi-time point analyses like those in the present study, it is particularly important that white matter tracts can be objectively and reliably defined.

Behaviorally, children improved their performance on arithmetic problem solving tasks after 2 months of intense math tutoring. Critically, individual behavioral gains after tutoring were positively correlated with FA changes in the left SLF-FT and to a lesser extent the right SLF-FP. Our findings provide the first evidence that math learning in children is characterized by dynamic changes in white matter integrity of specific white matter tracts linking the posterior association cortices with the frontal lobe.

### SLF fiber-tract identification in children

To our knowledge, the present study is the first to reliably identify distinct SLF tracts in children using automated query-language procedures that allow more precise and consistent quantification of white matter tracts across multiple time points. Crucially, our approach allows detailed specification of not just the start and end points of the tracts, but also the intermediate white matter regions through which the trajectory of the pathways courses. Such precision is important for reliably measuring longitudinal changes in the same tracts.

While prior cross-sectional studies have found an association between math performance and integrity of white matter tracts connecting frontal, parietal and temporal regions (Tsang et al. 2009; Van Beek et al. 2013; van Eimeren et al. 2008; Matejko et al. 2012; Navas-Sanchez et al. 2013), findings have been inconsistent with respect to the regional specificity of the effects. This may be partly due to the widely differing methods used to assess white matter integrity in each of these studies, each with their own advantages and disadvantages. For example, a drawback of voxel-based methods, the most widely used approach, is that they are carried out in a standard space and therefore depend on the accuracy of normalization procedures. Besides, the specific locus of effects could differ across participants with the consequence that the key effects might go undetected in whole-brain voxel-wise group results. Two recent studies have combined the whole-brain approach with atlas-based parcellation procedures, which allows extraction of tract-specific mean FA values in subject space (Kucian et al. 2013; Navas-Sanchez et al. 2013). Our approach was analogous to atlas-based parcellation of white matter tracts in that we extracted each participant’s mean FA in subject space using probabilistic tract maps. Yet, an advantage over the atlas-based method is that our probabilistic maps were created using the WMQL, which allowed greater specificity in terms of tract definition and provided age-specific template maps. Other studies have used a manual fiber tracking approach to examine FA in individually defined white matter regions (Tsang et al. 2009; Van Beek et al. 2013). While manual tractography takes into account inter-individual variations in size and shape of white matter tracts, it is also highly subjective. The advantage of our WMQL approach is that it provides great sensitivity and precision without the need for labor-intensive efforts and subjective judgments, an issue that is particularly important for fiber tracking across multiple time points in the same individuals.

Using this novel approach, we identified three SLF tracts following distinct trajectories in fronto-parietal, fronto-temporal and parieto-temporal white matter (Fig. 2): (1) the SLF-FP involved the fiber bundles contained in the frontal and parietal lobes connecting the frontal lobe with the supramarginal and inferior parietal cortex. This tract is composed of the section that Catani et al. (2007, 2005) refer to as the *anterior indirect segment*, and shows close correspondence to the SLF II and III in the SLF subdivision of Makris et al. (2005). (2) The SLF-FT involved the fiber bundles connecting the inferior frontal, middle frontal and precentral gyrus with the superior and middle temporal gyrus coursing through the parietal lobe (Catani et al. 2005, 2007; Makris and Pandya 2009). This tract is composed of what Catani et al. (2007, 2005) refer to as the *long direct segment,* and includes the fronto-temporal section of the SLF, or arcuate fasciculus as defined by Makris et al. (2005) and Makris and Pandya (2009). (3) The SLF-PT involved the fiber bundles contained in the temporal and parietal lobes connecting the temporal lobe with the inferior parietal convolutions. This tract is composed of the fasciculus that Catani et al. (2007, 2005) define as the *posterior indirect segment*, and the section of the inferior longitudinal fasciculus that innervates the inferior parietal lobule (Makris et al. 1999). Notably, the three major SLF tracts identified in the 7- to 9-year-old children that were included in our study are generally consistent with the SLF-fp, SLF-t and SLF-pt tracts previously identified in adults (Zhang et al. 2010). Our experiments of tract spatial overlap within subjects showed that the three sections of the SLF were highly reliable across the two time points. The lowest overlap values were on the left hemisphere. Particularly, one of the lowest overlap scores was for the left SLF-FT tract, which is consistent with our most significant findings on FA changes with tutoring. The high reliability of the DTI pipeline for the extraction of the FA_tract_ measure was shown by the intra-class coefficient value on the left and right CST, which was above 0.9 in both cases. As the CST was not expected to change with the tutoring administered to the subjects, this score indicates that the measure we use as a proxy of changes in the organization of white matter tissue on SLF tracts is reliably obtained with our longitudinal DTI data processing pipeline.

### Left fronto-temporal section of the SLF is a major locus of individual differences in learning-related white matter changes in children

Using the three major SLF tracts identified in each hemisphere, we found that the strongest relation between tutoring-related performance gains and changes in white matter integrity was in the left SLF-FT. This tract connects posterior temporal and lateral prefrontal cortices, coursing through the posterior parietal cortex, and is therefore well positioned to integrate symbolic, numerical, and control processing carried out by distributed brain regions (e.g., Arsalidou and Taylor 2011). Notably, additional analyses showed that brain–behavior correlations were present for three of the four behavioral sub-measures—accuracy on subtraction and RT on addition and subtraction—indicating that the brain–behavior correlations reflected general performance improvements and were not driven by one specific measure. Moreover, the brain–behavior correlation was still significant after partialling out FA from the neighboring tracts, indicating the anatomical specificity of the effect. In contrast to previous studies, which were mostly conducted in older children and adolescents, no significant brain–behavior relations were observed in the left SLF-FT before tutoring. While the lack of brain–behavior correlations before tutoring might be due to low power, the results suggest the intriguing possibility that in the narrow age-range of 3rd graders who comprised our sample, brain–behavior correlations may only be observed after extensive learning has taken place.

The SLF-FT as defined in the present study included the fronto-temporal section of the SLF, or arcuate fasciculus as described by Makris et al. (2005) and Makris and Pandya (2009). The tract also overlapped with portions of the SLF-II, which according to definitions by Makris et al. (2005), Schmamann and Pandya (2006) and Schmahmann et al. (2007) lies above and adjacent to the arcuate fasciculus. It is important to note that the exact functionality, origins and terminations of the SLF-FT, as well as its differentiation from other sections of the SLF are still under debate (Schmahmann and Pandya 2006; Dick and Tremblay 2012). Historically, the SLF-FT or arcuate fasciculus has been referred to as a language-related fiber bundle, providing a direct connection between Broca’s and Wernicke’s areas. In agreement with this view, voxel-based DTI analyses have demonstrated a link between language ability/dyslexia and white matter integrity in left temporo-parietal and frontal areas (e.g., Klingberg et al. 2000; Deutsch et al. 2005; Steinbrink et al. 2008; Nagy et al. 2004; Gold et al. 2007), which coincide with the arcuate fasciculus, as well as the neighboring superior corona radiata (Vandermosten et al. 2012b). Furthermore, the involvement of the left arcuate fasciculus to reading and language has been confirmed by fiber tracking studies in both children and adults (Vandermosten et al. 2012a; 2011, Yeatman et al. 2012). In contrast, anatomical tracing studies in monkeys suggest that the SLF-FT or arcuate fasciculus links posterior temporal and dorsolateral prefrontal cortex regions involved with spatial attention, sound localization, and monitoring of information in working memory (Schmahmann and Pandya 2006; Schmahmann et al. 2007), suggesting that the tract has a more domain-general function.

Our 8-week tutoring emphasized conceptual understanding of arithmetic using physical manipulatives, including a number line and physical objects, along with rapid retrieval of arithmetic facts, which draws on phonological processing. Behavioral studies have suggested that verbal, spatial and numerical processing are intimately related and play an important role in the learning and retrieving of basic arithmetic facts (Hubbard et al. 2005; Gunderson et al. 2012; Krajewski and Schneider 2009; Li and Geary 2013; De Smedt et al. 2010; De Smedt and Boets 2010). These observations suggest that the SLF-FT might contribute to learning by linking visuo-spatial, verbal, and executive control systems. How exactly SLF-FT plasticity associated with math learning impacts functional integration of spatial quantity-based processing (as supported by the dorsal parietal cortex) with phonological processing (as supported by the lateral temporal lobe and temporal-parietal cortex) remains to be further investigated.

Our findings have important implications for the remediation of math skills in children with math disabilities. Besides the left SLF-FT, plasticity in the right SLF-FP was also correlated with learning, but to a lesser extent. The identification of both left and right SLF tracts in relation to learning is of particular interest given previous reports of white matter deficits in children with math difficulties. Kucian and colleagues reported that children with developmental dyscalculia have reduced white matter integrity in both left and right SLF (Kucian et al. 2013). In addition, Rykhlevskaia et al. (2009) demonstrated that children with developmental dyscalculia have reduced white matter volume and FA in a right temporo-parietal cortex region, which included the SLF. Further studies with larger samples are needed to establish which of the three SLF tracts identified in our study are most impaired in developmental dyscalculia and to determine whether math tutoring can normalize deficits in SLF tracts (Butterworth et al. 2011).

Surprisingly, despite a positive correlation between FA changes and performance changes, there was no overall increase in FA in the entire group after training. Thus, some children showed increases in FA, while other children showed decreases. A similar profile of FA changes in the left arcuate fasciculus was observed in a longitudinal study of reading (Yeatman et al. 2012). Like in the present study, some children showed increases in FA with age, while others showed decreases, the average change across all children being close to zero (Yeatman et al. 2012). Despite this, Yeatman and colleagues found that the slope of FA changes was correlated with children’s reading abilities, illustrating, as in the present study, that the inter-individual variation in white matter changes is behaviorally relevant. To explain variability in FA changes, Yeatman and colleagues proposed a dual-process model characterized by white matter changes that reflect a combination of myelination (which increases FA) and axonal pruning (which decreases FA). Yet, it has been argued that changes in fiber organization, including changes in packing density, axon diameter or fiber crossings, may contribute to developmental and learning-related FA changes as well (Zatorre et al. 2012; Paus 2010; Beaulieu 2002). Furthermore, it is possible that FA changes are influenced by changes in crossing fiber tracts. For example, corona radiata fibers, as well as short cortico-cortial fibers, intermingle with sections of the SLF and arcuate fasciculus (Schmahmann and Pandya 2006), suggesting that increased myelination in corona radiata or short cortico-cortical fibers might lead to decreased FA in the tracts of interest. The precise mechanisms underlying developmental and learning-related changes in white matter integrity remain an important topic for future research.

### White matter plasticity in other academically relevant domains

Short-term learning-related plasticity of white matter pathways has also been observed in relation to reading and spelling training. A seminal study by Keller and Just (2009) demonstrated the effects of remedial reading instruction on white matter integrity in 8- to 12-year-old children with poor reading abilities. After 100 h of reading instruction, children showed increased FA in a region in the left anterior centrum semiovale, which connects the left superior frontal gyrus with the paracentral lobule. The same white matter region showed lower FA in poor readers prior to instruction, suggesting that remedial instruction may normalize anatomical connectivity in children with poor reading abilities. Similarly, Gebauer et al. (2012) found increased FA after a spelling intervention in spelling-impaired children. However, in contrast to the reading intervention study, changes were predominantly found in right hemisphere tracts including right superior corona radiata, internal capsule, and sections of the SLF, suggesting that acquisition of different skills may rely on different white matter tracts.

Interestingly, a cross-sectional study comparing a group of 10-year-old Chinese children who had received intensive abacus calculation training (>3 years) with a control group, showed increased FA in white matter tracts related to bimanual motor coordination and visuospatial processing (corpus callosum, occipito-temporal regions and right premotor cortex) (Hu et al. 2011). Although these results seem inconsistent with the present findings, the discrepancy between studies is in line with functional activation patterns during mental calculation in Chinese versus English speakers. That is, while native Chinese speakers preferentially employ a visuo-premotor network, native English speakers rely more on left perisylvian language regions (Tang et al. 2006). Taken together, these findings suggest that white matter plasticity depends on the skill and learning strategies that are employed. Future studies should further examine the specificity of white matter changes by comparing FA changes after different types of tutoring protocols in a randomized controlled design. Besides controlling for test–retest effects and maturational changes, such studies have the potential to further clarify the neuroanatomical pathways associated with children’s learning capacity across different academic disciplines and cognitive domains. They may also aid in the development of individualized learning interventions by providing a better understanding of the underlying mechanisms that drive learning and academic skill acquisition in different children.

## Conclusion

Using a well-validated, one-on-one math tutoring program, we demonstrated a critical relation between learning-induced gain in math abilities and white matter changes in 3rd grade children. Importantly, we used a novel automated fiber tracking algorithm to create child-specific probabilistic maps of the three major sections of the SLF. This method allowed us to objectively and reliably define white matter tracts across multiple time points. Our results extend previous findings of individual differences in white matter associated with math skills, and point to the important role of plasticity in left hemisphere perisylvian connections for math learning. More generally, our findings provide important new insights into experience-dependent white matter changes in childhood, suggesting that inter-individual variability in white matter integrity changes dynamically with learning and development.

## Electronic supplementary material

Below is the link to the electronic supplementary material.

Supplementary material 1 (PDF 64 kb)

Supplementary material 2 (PDF 77 kb)

Supplementary material 3 (PDF 59 kb)

Supplementary material 4 (PDF 1483 kb)

## Footnotes

1
http://tract-querier.readthedocs.org.
