# Supplementary material for: Plasticity of left perisylvian white-matter tracts is associated with individual differences in math learning
Source: Brain Struct Funct. 2015 Jan 21;221(3):1337–51. doi: 10.1007/s00429-014-0975-6 (PMC4819785; doi:10.1007/s00429-014-0975-6)
Supplement: Supplementary file 1 — Supplementary material 1 (PDF 64 kb) [file 429_2014_975_MOESM1_ESM.pdf]

Online Resource 1: Tutoring procedures

Plasticity of left perisylvian white-matter tracts is associated with individual differences in math learning

Dietsje Jolles, Demian Wassermann*, Ritika Chokhani, Jennifer Richardson, Caitlin Tenison, Roland Bammer, Lynn Fuchs, Kaustubh Supekar, Vinod Menon*

Address for correspondence: d.d.jolles@fsw.leidenuniv.nl; menon@stanford.edu

** contributed equally to this work*

Submitted to Brain Structure and Function

Tutoring procedures

Each child completed a one-on-one two-month intervention program adapted from Math Wise (Fuchs et al. 2009) and Galaxy Math (Fuchs et al. 2013), which combined conceptual instruction with speeded procedural practice of simple addition and subtraction problems (Supekar et al. 2013). Similar to MathWise and Galaxy Math, the tutoring involved a total of 15-20 hours of training, but it was condensed to 8/9 weeks with longer lessons. The tutoring consisted of 22 lessons of increasing difficulty. Lessons 1 through 4 reviewed adding and subtracting 0, 1, and 2, as well as low ties (from 1+1 to 6+6 and corresponding subtraction facts e.g., 12-6). These lessons also taught the commutative property of addition (i.e., changing the order of the operands does not change the sum), as well as the additive identity property of zero (i.e., adding zero does not change the number's value). They also introduced the children to math manipulatives (i.e., a number line and blocks in a circle). Lessons 5 and 6 taught the "min strategy" for counting answers to addition problems (i.e., start with the larger number and count up with the smaller number) and the "missing addend strategy" for

counting answers to subtraction problems (i.e., start with the smaller number count up to the larger number) (Fuchs et al. 2009). During lesson 7 to 22, children practiced with progressively difficult problems. They started out with all problems in the 5 set (addition problems summing to 5 and subtraction problems with 5 as the minuend). By the end of tutoring, they learned addition problems that summed to 18, and their corresponding subtraction problems. All lessons followed the same structure: (1) warm-up flashcards to review previously trained math problems; (2) number knowledge review, including the use of manipulatives and the counting strategies; (3) a lesson worksheet to introduce the new math problems; (4) a math game, (5) computerized flashcards combining the current and previous lessons' material (programmed in Scratch; Resnick et al. 2009), (6) a physical flashcard game, and (7) a review worksheet on that day's problem set. Since scanning occurred only on weekends, children who completed lesson 22 early in the week took part in (maximally 2) additional review sessions.

Fuchs LS, Geary DC, Compton DL, Fuchs D, Schatschneider C, Hamlett CL, DeSelms J, Seethaler PM, Wilson J, Craddock CF, Bryant JD, Luther K, Changas P (2013) Effects of first-grade number knowledge tutoring with contrasting forms of practice. J Educ Psychol 105 (1):58-77

Fuchs LS, Powell SR, Seethaler PM, Cirino PT, Fletcher JM, Fuchs D, Hamlett CL, Zumeta RO (2009) Remediating number combination and word problem deficits among students with mathematics difficulties: A randomized control trial. J Educ Psychol 101 (3):561

Resnick M, Maloney J, Monroy-Hernandez A, Rusk N, Eastmond E, Brennan K, Millner A, Rosenbaum E, Silver J, Silverman B (2009) Scratch: Programming for all. Communications of the ACM 52 (11):60-67

Supekar K, Swigart AG, Tenison C, Jolles DD, Rosenberg-Lee M, Fuchs L, Menon V (2013) Neural predictors of individual differences in response to math tutoring in primary-grade school children. Proc Natl Acad Sci U S A 110 (20):8230-8235. doi:10.1073/pnas.1222154110
