# Supplementary material for: Plasticity of left perisylvian white-matter tracts is associated with individual differences in math learning
Source: Brain Struct Funct. 2015 Jan 21;221(3):1337–51. doi: 10.1007/s00429-014-0975-6 (PMC4819785; doi:10.1007/s00429-014-0975-6)
Supplement: Supplementary file 2 — Supplementary material 2 (PDF 77 kb) [file 429_2014_975_MOESM2_ESM.pdf]

Online Resource 2: Supplementary Tables

Plasticity of left perisylvian white-matter tracts is associated with individual differences in math learning

Dietsje Jolles, Demian Wassermann*, Ritika Chokhani, Jennifer Richardson, Caitlin Tenison, Roland Bammer, Lynn Fuchs, Kaustubh Supekar, Vinod Menon*

Address for correspondence: d.d.jolles@fsw.leidenuniv.nl; menon@stanford.edu

** contributed equally to this work*

Submitted to Brain Structure and Function

Table 1 Mean scores and standard deviations on neuropsychological measures

	Mean	*SD*	n
Age	8.55	0.39	
Gender	11F / 7M		
IQ			
Verbal IQ	105.67	16.57	18
Performance IQ	104.17	11.26	18
Full-scale IQ	105.56	12.11	18
Math			
WIAT-II Numerical Operations	101.67	14.04	18
WIAT-II Mathematical Reasoning	105.39	12.46	18
Keymath Numeration	9.88	2.12	17
Keymath Algebra	9.47	2.65	17
Reading			
WIAT-II Word Reading	104.94	9.91	18
WIAT-II Reading Comprehension	108.00	10.87	18
Working Memory			
WMTB-C Digit Recall	99.89	15.99	18
WMTB-C Backward Digit Recall	89.11	16.40	18
WMTB-C Block Recall	91.94	11.62	18
WMTB-C Count Recall	83.41	16.82	17

WIAT = Wechsler Individual Achievement Test; WMTB-C = Working Memory Test Battery for Children

Table 2 FA changes in each of the six tracts of interest

Tracts	Repeated Measures ANOVA
Left SLF-FP	$F(1,17) = 0.007$, $p = .933$
Left SLF-FT	$F(1,16) = 1.972$, $p = .179$^
Left SLF-PT	$F(1,17) = 0.325$, $p = .576$
Right SLF-FP	$F(1,17) = 0.005$, $p = .943$
Right SLF-FT	$F(1,17) = 0.819$, $p = .378$
Right SLF-PT	$F(1,17) = 0.424$, $p = .524$

^ One outlier was excluded from this analysis (with FA change > 3 *SD*s).

Table 3 Correlation between performance changes and FA changes in each of the six tracts of interest

Tracts	Pearson Correlation	*p*	n
Left SLF-FP	-.010	.969	18
Left SLF-FT	.630	.007**	17^
Left SLF-PT	-.259	.299	18
Right SLF-FP	.466	.051	18
Right SLF-FT	-.269	.281	18
Right SLF-PT	-.302	.223	18

** $p < .01$; ^ One outlier was excluded from this analysis (with FA change > 3 *SD*s). A non-parametric correlation including the outlier was still significant ($p < .05$).

Table 4 Correlation between FA in the SLF-FT before tutoring and math ability

**Tracts**	**Pearson Correlation**	***p***	**n**
WIAT-II Numerical Operations	-.216	.405	17
WIAT-II Mathematical Reasoning	-.124	.636	17
Keymath Numeration	-.077	.777	16
Keymath Algebra	-.225	.402	16
Efficiency	-.014	.959	17

One outlier was excluded from all analyses (with FA change > 3 *SD*s).
