# Supplementary material for: Plasticity of left perisylvian white-matter tracts is associated with individual differences in math learning
Source: Brain Struct Funct. 2015 Jan 21;221(3):1337–51. doi: 10.1007/s00429-014-0975-6 (PMC4819785; doi:10.1007/s00429-014-0975-6)
Supplement: Supplementary file 3 — Supplementary material 3 (PDF 59 kb) [file 429_2014_975_MOESM3_ESM.pdf]

Online Resource 3: White Matter Query Language (WMQL) Script

Plasticity of left perisylvian white-matter tracts is associated with individual differences in math learning

Dietsje Jolles, Demian Wassermann*, Ritika Chokhani, Jennifer Richardson, Caitlin Tenison, Roland Bammer, Lynn Fuchs, Kaustubh Supekar, Vinod Menon*

Address for correspondence: d.d.jolles@fsw.leidenuniv.nl; menon@stanford.edu

** contributed equally to this work*

Submitted to Brain Structure and Function

White Matter Query Language (WMQL) Script

```
#Obtain all parcellation definitions from Freesurfer
import FreeSurfer.qry

middlefrontalgyrus.side |= (
                              rostralmiddlefrontal.side or
                              caudalmiddlefrontal.side
                   )
inferiorfrontalgyrus.side |= (
                              parsopercularis.side or
                              parstriangularis.side or
                              parsorbitalis.side
                 )
orbitofrontalgyrus.side |= (
                              lateralorbitofrontal.side or
                              medialorbitofrontal.side
                 )
frontal.side |=  (
                              superiorfrontal.side or
                              middlefrontalgyrus.side or
                              inferiorfrontalgyrus.side or
                              orbitofrontalgyrus.side or
                              frontalpole.side or
                              precentral.side or
                    paracentral.side
       )


temporal.side |= (
```

```
        (entorhinal.side or parahippocampal.side or temporalpole.side or
         fusiform.side or superiortemporal.side or middletemporal.side or
         inferiortemporal.side or transversetemporal.side or bankssts.side)
      )


parietal.side |= postcentral.side or precuneus.side or supramarginal.side or superiorparietal.side
         or inferiorparietal.side

superior_parietal_lobule.side |= postcentral.side or precuneus.side or superiorparietal.side
inferior_parietal_lobule.side |= supramarginal.side or inferiorparietal.side

subcortical.side |= VentralDC.side or Thalamus_Proper.side or Pallidum.side or Putamen.side
         or Amygdala.side or Brain_Stem or choroid_plexus.side


inferior_frontal_gyrus.side |= parsopercularis.side or parstriangularis.side or parsorbitalis.side

middle_frontal_gyrus.side |= caudalmiddlefrontal.side or rostralmiddlefrontal.side

#Fronto-Parietal SLF
SLF_FP.side = (endpoints_in(inferiorparietal.side) or endpoints_in(supramarginal.side)) and
       endpoints_in(frontal.side) and only(parietal.side or frontal.side or
       unsegmentedwhitematter.side)

#Parieto-Temporal SLF
SLF_PT.side = endpoints_in(inferiorparietal.side) and endpoints_in(temporal.side) and
       only(parietal.side or temporal.side or unsegmentedwhitematter.side)

#Fronto-Temporal SLF
SLF_FT.side = (
         (inferior_frontal_gyrus.side or middle_frontal_gyrus.side or precentral.side) and
        (superiortemporal.side or middletemporal.side) and not medial_of(supramarginal.side)
         not in hemisphere.opposite
         not in temporalpole.side not in frontalpole.side not in subcortical.side
         not in rostralmiddlefrontal.side not in lateralorbitofrontal.side
         not in parstriangularis.side not in superiorfrontal.side not in parsopercularis.side
       )
```
