# Supplementary material for: Plasticity of left perisylvian white-matter tracts is associated with individual differences in math learning
Source: Brain Struct Funct. 2015 Jan 21;221(3):1337–51. doi: 10.1007/s00429-014-0975-6 (PMC4819785; doi:10.1007/s00429-014-0975-6)
Supplement: Supplementary file 4 — Supplementary material 4 (PDF 1483 kb) [file 429_2014_975_MOESM4_ESM.pdf]

Online Resource 4: Supplementary Figures

# Plasticity of left perisylvian white-matter tracts is associated with individual differences in math learning

*Dietsje Jolles*, Demian Wassermann*, Ritika Chokhani, Jennifer Richardson, Caitlin Tenison, Roland Bammer, Lynn Fuchs, Kaustubh Supekar, Vinod Menon*

Address for correspondence: d.d.jolles@fsw.leidenuniv.nl; menon@stanford.edu

** contributed equally to this work*

Submitted to Brain Structure and Function

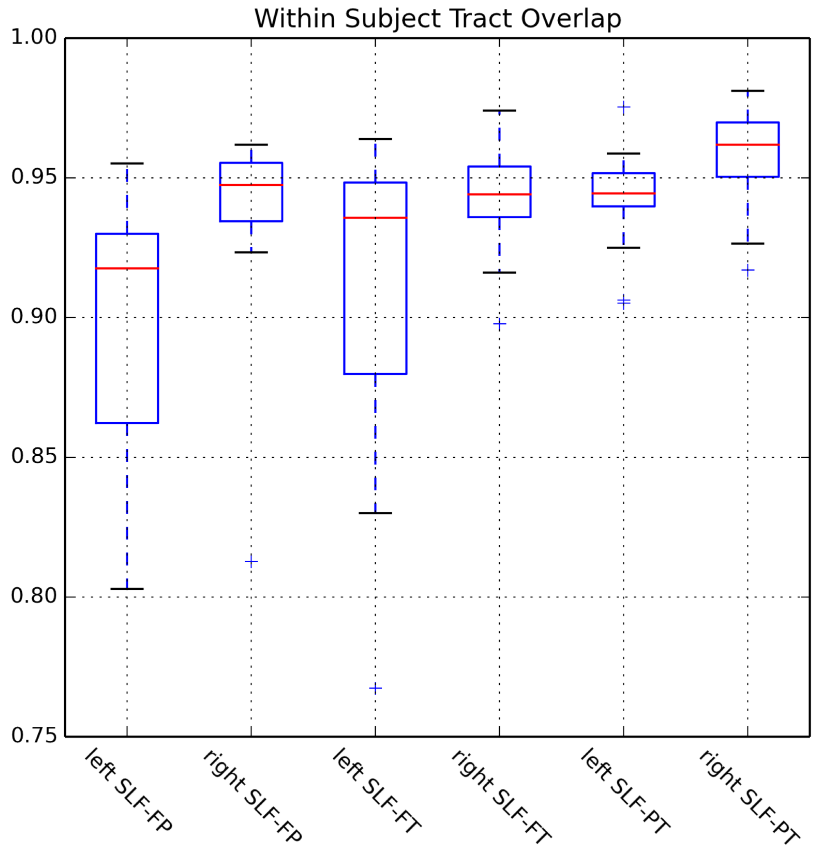


**Supplementary Fig. 1 High reliability of tract-identification.** From left to right, boxplots represent reliability of the left and right fronto-parietal section of the SLF (SLF-FP), fronto-temporal section of the SLF (SLF-FT), and parieto-temporal section of the SLF (SLF-PT). The within-subject overlap of the SLF tracts was quantified using the

Bhattacharyya coefficient, which has a maximum value of 1 (perfect overlap) and a minimum value of 0 (no overlap). An overlap of about 0.8 was considered an almost perfect overlap. The maximum average overlap was found for the right SLF-PT and the minimum overlap for the left SLF-FP.

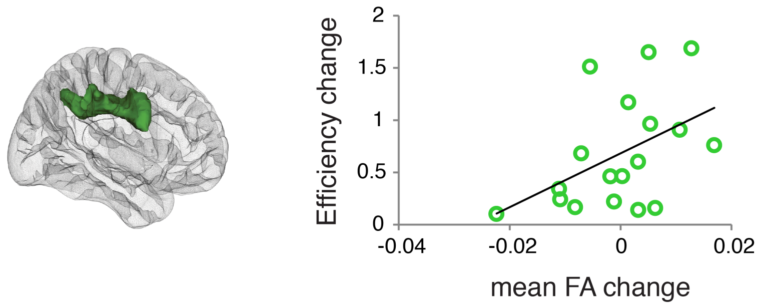


**Supplementary Fig. 2 Performance gains are marginally correlated with FA changes in the fronto-parietal part of the right superior longitudinal fasciculus (SLF-FP)** Performance efficiency was assessed using a composite of standardized accuracy and reaction time (RT) scores on addition and subtraction tasks (N = 18). FA = fractional anisotropy; $p = .051$
